# Antinociceptive Effect of *Dendrobii caulis* in Paclitaxel-Induced Neuropathic Pain in Mice

**DOI:** 10.3390/life13122289

**Published:** 2023-11-30

**Authors:** Keun Tae Park, Yong Jae Jeon, Hyo In Kim, Woojin Kim

**Affiliations:** 1 Department of Physiology, College of Korean Medicine, Kyung Hee University, Seoul 02453, Republic of Korea; cerex@naver.com (K.T.P.); ad2353@naver.com (Y.J.J.); 2 Korean Medicine-Based Drug Repositioning Cancer Research Center, College of Korean Medicine, Kyung Hee University, Seoul 02447, Republic of Korea; 3 Department of Surgery, Beth Israel Deaconess Medical Center, Harvard Medical School, Boston, MA 02115, USA; hkim21@bidmc.harvard.edu

**Keywords:** *Dendrobii caulis*, *Dendrobium officinale*, paclitaxel, transient receptor potential vanilloid 1

## Abstract

Paclitaxel-induced neuropathic pain (PINP) is a serious adverse effect of chemotherapy. *Dendrobii caulis* (*D. caulis*) is a new food source used as herbal medicine in east Asia. We examined the antinociceptive effects of *D. caulis* extract on PINP and clarified the mechanism of action of transient receptor potential vanilloid 1 receptor (TRPV1) in the spinal cord. PINP was induced in male mice using multiple intraperitoneal injections of paclitaxel (total dose, 8 mg/kg). PINP was maintained from D10 to D21 when assessed for cold and mechanical allodynia. Oral administration of 300 and 500 mg/kg *D. caulis* relieved cold and mechanical allodynia. In addition, TRPV1 in the paclitaxel group showed increased gene and protein expression, whereas the *D. caulis* 300 and 500 mg/kg groups showed a significant decrease. Among various substances in *D. caulis*, vicenin-2 was quantified by high-performance liquid chromatography, and its administration (10 mg/kg, i.p.) showed antinociceptive effects similar to those of *D. caulis* 500 mg/kg. Administration of the TRPV1 antagonist capsazepine also showed antinociceptive effects similar to those of *D. caulis*, and *D. caulis* is thought to exhibit antinociceptive effects on PINP by modulating the spinal TRPV1.

## 1. Introduction

Chemotherapy can cause peripheral neuropathic pain, leading to discontinuation or dose reduction [1]. Highly effective anticancer drugs, such as oxaliplatin, vincristine, cisplatin, and paclitaxel, can cause peripheral neuropathic pain [2,3]. Paclitaxel, discovered and isolated from *Taxux brevifolia* [4,5], is an anticancer drug used for breast, lung, and ovarian cancer [6]. Although various side effects such as bone marrow toxicity, myalgia, and arthralgia have been reported, chronic and acute peripheral neuropathy is one of the most serious side effects [7]. Although the prevalence of paclitaxel-induced neuropathic pain (PINP) is 60% [8], PINP treatment remains unsatisfactory despite numerous studies. Furthermore, no specific drugs are available for treatment or prevention [9].

*D. caulis* is a widely recognized traditional tonic in China, used not only as a supplement for patients but also in food, and is native to China and southeast Asian countries [10,11]. *D. caulis* is the dried stem of *Dendrobium officinale* (*D. officinale*). *Fengdou* (the twisted dried stem part) and its powder are sold in their representative forms in the market and used in various ways, such as making soups, medicinal liquor, and teas [12]. Although there are no reports on *D. caulis*, several studies have reported curative effects of *D. officinale*. Administration of 200 mg/kg *D. officinale* improved intestinal barrier function and strengthened the antitumor immune response in a colorectal cancer mouse model [13]. Additionally, in an acute colitis model, administration of 200 mg/kg of polysaccharide from *D. officinale* alleviated liver damage by increasing antioxidant enzyme activity and downregulating the tumor necrosis factor-α (TNF-α) signaling pathway [14]. In a rat model of type 2 diabetes, administration of 160 mg/kg improved oxidative stress, inflammation, and liver lipid accumulation [15]. Studies have shown that the glucomannan fraction of *D. officinale* attenuates intestinal damage in colitis mice and regulates intestinal mucosal immunity [16]; in cell studies, it is regulated through the toll-like receptor 4/nuclear factor kappa-light-chain-enhancer of activated B cell (TLR4/NF-kB) signaling pathways in gastric epithelial cells (GES-1) [17].

Chemical analysis of *D. caulis* has shown that flavonoids, phenols, and polysaccharides are the major compounds isolated from the stem [18]. Flavonoids are important compounds in *D. caulis* and have potential anticancer, anti-inflammatory, and antioxidant properties. In particular, *D. caulis* is rich in flavonoid C-glycosides [18], and as a result of high-performance gel permeation chromatography analysis, a total of 26 compounds were identified, of which 14 flavonoids including rutin and vicenin-2 were reported as major substances [19].

Members of the transient receptor potential (TRP) family exist in ion channels at the terminals of nociceptors which act as molecular transducers, where many stimuli depolarize neurons [20,21]. TRP channels constitute a unique superfamily of ion channels and are related to the voltage-gated Ca^2+^, K^+^, and Na^+^ superfamily. The transient receptor potential vanilloid receptor (TRPV) subfamily consists of channels involved in thermosensitivity and nociception (TRPV 1-4) and channels involved in Ca^2+^ uptake/reuptake (TRPV 5–6) [22]. Among TRP channels, the most studied is TRPV1. TRPV1 is expressed in sensory ganglia such as the dorsal root ganglia (DRG), vagal and trigeminal ganglia, and in small sensory Aδ- and C fibers which contain various neuropeptides such as calcitonin-related peptide and substance P [23,24,25,26,27,28]. TRPV1 is also found in central nervous system and non-nervous tissues such as the kidney, mast cells, smooth muscle, bladder, and lung [29,30,31,32].

Members of the transient receptor family of TRP channels reportedly contribute to PINP induction [33,34]. TRPV4 and transient receptor potential cation channel subfamily melastatin member 8 (TRPM8) showed sensitivity in the DRG neurons involved in PINP, whereas TRPV1 was considered a mediator of allodynia [35,36,37]. Additionally, oral administration of HC-030031, a selective TRPA1 antagonist, has been reported to be responsible for the mechanical hypersensitivity observed in neuropathic and inflammatory pain models [38]. On the other hand, in humans, when TRPV1 is activated in muscles, patients treated with paclitaxel experience cramping or deep pain [39]. TRPV1 activation is closely related to what chemotherapy-induced neuropathy patients experience [40,41]. In patients, TRPV1 activation causes a burning sensation [42] in skin nociceptors and pain in deep tissue nociceptors [43]. Paclitaxel sensitizes and activates TRPV1 function, and TRPV1 antagonists exert analgesic effects in chemotherapy-induced neuropathy [34]. Similarly, cisplatin or bortezobim treatment increased the expression of TRPV1 in the spinal cord and dorsal root ganglion of mice [44,45]. Paclitaxel interacts directly with TRPV1 channels to acutely stimulate signaling and also produce long-term channel desensitization changes [46]. Parallel studies have shown that oxaliplatin also sensitizes TRPV1, which is mediated by the G-protein-coupled receptor G2A [47].

In the present study, we investigated the effects of *D. caulis* extract on PINP. Subsequently, changes in the gene and protein expression of TRPV1 in the spinal cord caused by paclitaxel were confirmed. In addition, the effect of *D. caulis* and vicenin-2, its active ingredient, on TRPV1 was demonstrated, and their antinociceptive effect on PINP was evaluated.

## 2. Materials and Methods

### 2.1. Animals

Six-week-old male C57BL/6J mice were purchased from Daehan Biolink (Daejeon, South Korea). The mice were kept under controlled conditions for one week to adapt. The lighting cycle was adjusted to 12 h light/12 h dark, and constant temperature (23 ± 2 °C) and humidity (65 ± 5%) were maintained. After adaptation, six animals were randomly assigned to each group. During the experimental period, the mice were provided with a standard diet and freely available water. All experimental protocols were approved by the Kyung Hee University Animal Care and Use Committee (approval NO. KHUASP-23-223, 12 April 2023). In total, 80 mice were used and all experiments were conducted in accordance with the guidelines of the International Association for the Study of Pain [48].

### 2.2. Dendrobii caulis Extract Preparation

Dried *D. caulis* was purchased from Xiuzheng Pharmaceutical Group Co., Ltd. (Hangzhou, China). *D. caulis* was extracted by 25% ethanol and reflux extraction for 6 h at 80 °C. The extract was concentrated under reduced pressure at 60 °C after filtering the residue through filter paper. The extract was dried using an evaporator and then frozen using a lyophilizer to obtain the extract powder. *D. caulis* was diluted in phosphate-buffered saline (PBS) and administered orally or intrathecally to mice (Specimen N. KWJ-0003).

### 2.3. Paclitaxel, D. caulis, Vicenin-2, and Capsazepine Administration

**Figure 1:**
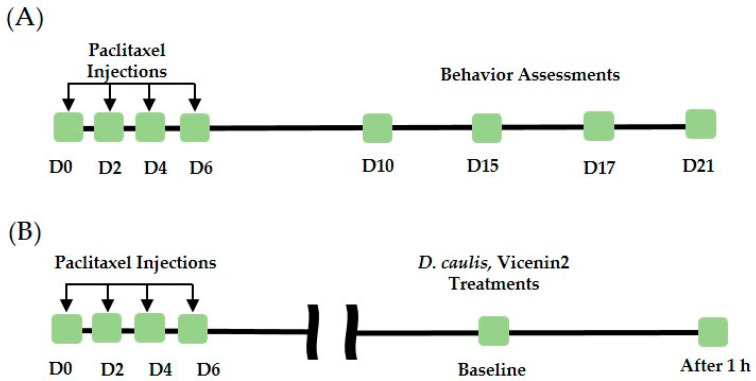
The effect of multiple intraperitoneal paclitaxel injections in mice. Schedule of behavior tests conducted to assess the cold and mechanical allodynia after paclitaxel injection (**A**). Antinociceptive effect evaluation time progress (**B**).

Paclitaxel (Sigma-Aldrich, St. Louis, MO, USA) was dissolved in a 1:1 mixture of Cremophor EL (Sigma-Aldrich) and ethanol at a concentration of 6 mg/mL. Paclitaxel was diluted to 0.2 mg/mL in PBS, and 200 μL was intraperitoneally administered to mice. In the vehicle group, the same volume of a 1:1 solution of Cremophor EL and ethanol was administered via the same route. Paclitaxel and vehicle solutions were injected intraperitoneally four times every other day (D0, 2, 4, and 6) at a dose of 2 mg/kg (Figure 1). The *D. caulis* extract was administered orally using a sonde (Jungdo-BNP, Seoul, Republic of Korea) at different doses (100, 300, and 500 mg/kg in distilled PBS). Vicenin-2 (Sigma-Aldrich, St. Louis, MO, USA) was dissolved in 10% dimethyl sulfoxide (DMSO) and 100 μL was administered intraperitoneally at doses of 1 and 10 mg/kg. In the intrathecal injection administration experiment, 10 μg/mouse of capsazepine, a TRPV1 antagonist, was dissolved in 10% DMSO, and *D. caulis* was dissolved in doses of 0.1 and 1 mg/mouse in PBS, and the administered volume was 5 μL. *D. caulis*, vicenin-2, and capsazepine were administered between D10 and D21, the period when pain was maintained, and a behavioral assessment was conducted 1 h after administration.

### 2.4. Behavioral Tests

The acetone drop and von Frey filament methods were used to measure paclitaxel-induced cold and mechanical allodynia. Before the assessment, the mice were placed in a chamber made of steel wire for 30 min for acclimatization. The acetone drop method evaluated behavior by spraying 10 μL of acetone on the hind paw. The numbers of withdrawals, flinches, and licking reactions were recorded for 30 s [49]. The results show the average value of the ‘of Response’ measured [50,51]. To summarize the mechanical allodynia method, the mechanical allodynia test was started by selecting a strength of 3.22 among several von Frey filaments. The filament was pressed against the mid-plantar part of the hind paw for 2–3 s until it bent. If there was a pain response to the stimulation, a filament of strength one level lower was applied. If there was no pain response, pressure was applied using a filament with strength one level higher [52]. The pain response was marked with an X, otherwise, it was marked with an O. The ‘50% threshold value’ in the result refers to Chaplan’s calculation and Dixon’s up-down method [53,54]. After the behavioral evaluation, the mice were anesthetized with isoflurane. The mouse heart was perfused with PBS to wash the entire tissue, and lumbar spinal cord sections 4–5 were extracted. PCR and Western blotting were performed on the extracted tissues.

### 2.5. Quantitative Real-Time Polymerase Chain Reaction (qRT-PCR)

Total ribonucleic acid (RNA) was extracted from spinal tissues using an RNA extraction kit (AccuPrep, Bioneer, Daejeon, Republic of Korea). RNA samples were quantified using Nanodrop (Thermo Scientific, Waltham, MA, USA), and RT-PCR was performed on samples with an OD260/OD280 ratio higher than 2.0. cDNA was synthesized by incubating 0.5 μg of total RNA with the cDNA synthesized kit (Maxime RT Premix, Intron Biotechnology, Seongnam-Si, Republic of Korea), according to the protocol. qPCR amplification of cDNA was performed using a real-time SYBR kit (SensiFAST SYBR, Bio-Rad, Hercules, CA, USA) and CFX Real-Time PCR (Bio-Rad, Hercules, CA, USA). The real-time PCR condition was as follows: one cycle of 95 °C for 5 min, followed by 40 cycles of 95 °C for 20 s, 57 °C for 20 s, and 72 °C for 20 s. The primer sequences used were as follows: Glyceraldehyde 3-phosphate dehydrogenase (*Gapdh*) forward 5′-GTC GTG GAG TCT ACT GGT GTC TTC-3′ and reverse 5′-GTC ATC ATA CTT GGC AGG TTT CTC-3′, and transient receptor potential vanilloid 1 (*TRPV1*) forward 5′-GGC TGT CTT CAT CAT CCT GCT GCT-3′ and reverse 5′-GTT CTT GCT CTC CTG TGC GAT CTT GT-3′. GAPDH was used to normalize the amount of RNA in each sample, and a specific detection threshold (Ct) was selected to calculate the fluorescence produced by amplification. Relative gene expression compared to the control was quantified using the 2^−ΔΔCt^ method [55] with the average Ct value of each gene, and the expression value of the reference group was expressed as 1.

### 2.6. Western Blot

Western blotting was performed to analyze the protein expression of the TRPV1 receptor. Radioimmunoprecipitation assay buffer was added to liquefy the tissue, which was then centrifuged at 13,000 rpm for 10 min, and the supernatant was used for Western blotting. The protein concentration in the supernatant was quantified using a protein assay kit (Bradford protein assay kit, Bio-rad, Hercules, CA, USA). Then, 20 μg of protein was loaded to sodium dodecyl sulfate–polyacrylamide gel electrophoresis, following which the protein bands were transferred to nitrocellulose membrane. After blocking with 5% non-fat skim milk in tris-buffered saline with tween 20 buffer, the blocked membrane was incubated with primary antibodies overnight at 4 °C. Actin and TRPV1 proteins were detected using rabbit polyclonal anti-actin antibodies (1:1000, cat. PA1-183, Invitrogen, Waltham, MA, USA) and rabbit polyclonal anti-TRPV1 (1:1000, cat. NB100-1617, Novus Biologicals, Littleton, CO, USA), respectively. The membrane was washed with PBS-T and then further incubated with secondary antibody (horseradish peroxidase-conjugated anti-rabbit antibody,1:5000, cat. 31460, Thermo Scientific, Waltham, MA, USA) for 2 h at room temperature. Protein bands were detected using a chemiluminescence detection kit (D-Plus ECL Femto System, Hwaseong, Republic of Korea) and Davinch-Chemi software (Young-Hwa Science, Daejeon, Republic of Korea).

### 2.7. HPLC Analysis of Rutin and Vicenin-2 in D. caulis Extract

*D. caulis* was analyzed using high-performance liquid chromatography (HPLC) with a UV detector (Agilent 1260, Santa Clara, CA, USA). The conditions for the rutin, vicenin-2, and *D. caulis* extract analyses are listed in Table 1. Stock solutions of rutin and vicenin-2 were prepared in methanol. The rutin and vicenin-2 standard were purchased from Sigma-Aldrich (St. Louis, MO, USA) and Chemface (Wuhan, China), respectively. For both rutin and vicenin-2, five dilutions were prepared and analyzed. A total of 100 mg of *D. caulis* was ultrasonically extracted (4 °C, 60 min) using 1 mL of ethanol. After centrifuging (4 °C, 13,000 rpm, 30 min) the diluted solution, the supernatant was filtered through a 0.4 μm filter and used for analysis.

### 2.8. The Effect of Vicenin-2 on Caco-2 Cell and RAW 264.7 Cell Lines Measured by 3-(4,5-Dimethyl Thazolk-2-yl)-2,5-Diphenyl Tetrazolium Bromide (MTT) Viability Assay

Cell viability was evaluated in RAW 264.7 and Caco-2 cells obtained from the Korea Cell Line Bank (ATCC). Raw 274.7 cell lines were grown in Dulbecco’s modified Eagle’s medium supplemented with 10% fetal bovine serum (FBS), and Caco-2 cell lines were grown in Eagle’s minimum essential medium (MEM) supplemented with 10% FBS. Cultures was kept in a humidified atmosphere with 5% CO_2_ at 37 °C. The viability of cells were measured using the MTT assay. In a 96-well plate, 1 × 10^4^ cells, suspended to 200 μL of growth medium, were seeded for 24 h. After incubation, each concentration of vicenin-2 previously dissolved in DMSO was treated for 24 h. To evaluate the viability, the medium was discolored for an MTT working solution and incubated for 1 h at 37 °C. The formazan formed in the MTT assay was dissolved in DMSO and the optical density was measured at 540 nm using a microplate reader. The results were expressed as a percentage based on the control (untreated cells) group.

### 2.9. Statistical Analysis

The statistical analysis and graph work were performed using Prism GraphPad (version 9.0., Graphpad Software Inc., Boston, MA, USA). All data are presented as mean ± SD. Statistical analyses were performed using an unpaired *t*-test for the data shown in paclitaxel-induced pain experiment, whereas the data in *D. caulis*, vicenin-2 and antagonist administration experiments were analyzed using a paired *t*-test. In addition, statistical analyses were performed using one-way ANOVA followed by Tukey’s test for gene and protein expressions and cell viability analysis. A level of *p* < 0.05 was considered significant.

## 3. Results

### 3.1. Multiple Paclitaxel Injections Induce Cold and Mechanical Allodynia

**Figure 2:**
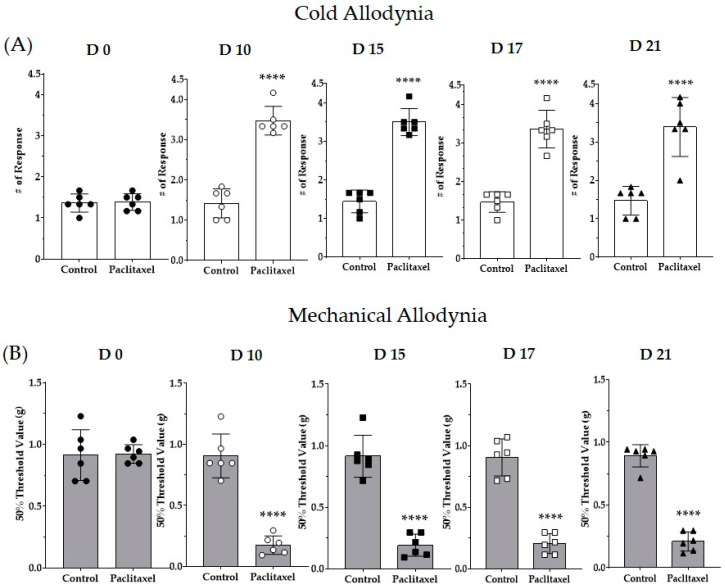
Pain assessment via multiple administrations of paclitaxel in mice. Paclitaxel was administered intraperitoneally at 2 mg/kg every 2 days (D0, D2, D4, and D6), and cold and mechanical allodynia were evaluated using the acetone drop method and the von Frey filament method. Behavioral assessment was conducted on D0, D10, D15, D17, and D21. Behavioral changes observed in the mice were recorded, calculated, and expressed as cold (**A**) and mechanical allodynia (**B**). The black circle, white circle, black square, white square, and black triangle represent D0, D10, D15, D17, and D21, respectively. N = 6 for each group; **** *p* < 0.0001 vs. control group with unpaired *t*-test.

Several studies have reported that multiple paclitaxel injections induce cold and mechanical allodynia in mice [56,57,58]. Paclitaxel was injected four times (D0, 2, 4, and 6, total 8 mg/kg), and the vehicle (1:1 ratio of Cremophor EL to ethanol) was administered to the control group. Significant pain was induced in both cold allodynia results obtained using the acetone drop method and mechanical allodynia results obtained using the von Frey filament method (Figure 2A,B). The results showed that paclitaxel injection increased the response to cold pain and decreased the threshold for mechanical stimulation from D10 to D21. All subsequent behavioral assessments were evaluated between D10 and D21.

### 3.2. Single Oral Administration of D. caulis in Cold and Mechanical Allodynia

**Figure 3:**
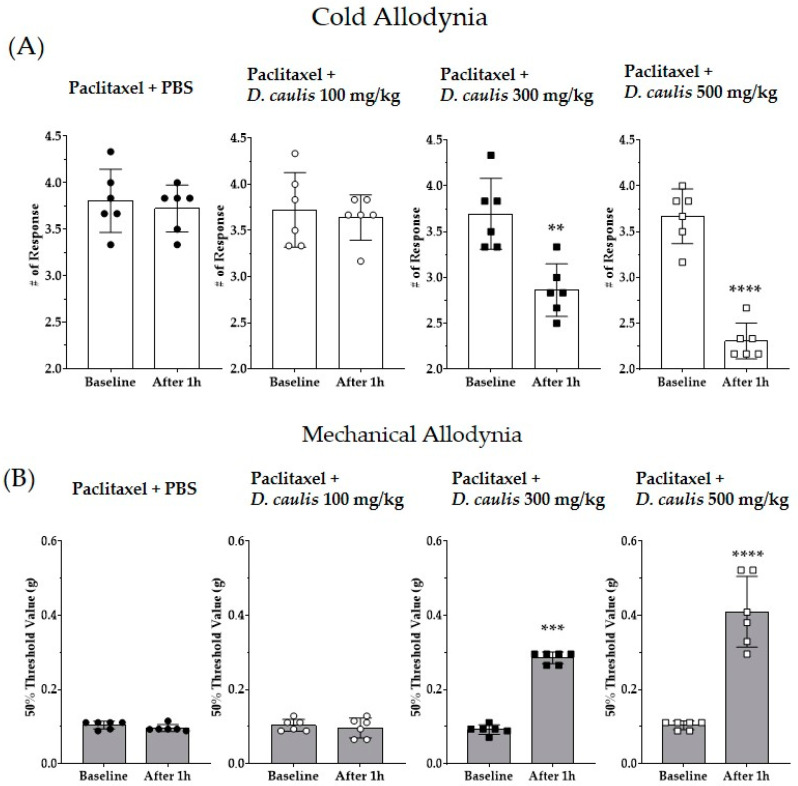
Effect of oral administration of *D. caulis* on cold and mechanical allodynia (**A**,**B**). Behavioral assessments were performed before *D. caulis* administration and 1 h after administration via gavage at concentrations of 100, 300, and 500 mg/kg. PBS was used as a vehicle for *D. caulis* and *D. caulis* or PBS was orally administered. The black circle, white circle, black square, and white square represent PBS control group, *D. caulis* 100 mg/kg group, 300 mg/kg, 500 mg/kg group, respectively. N = 6 for each group; ** *p* < 0.01, *** *p* < 0.001, and **** *p* < 0.0001 vs. control group with paired *t*-test.

The 25% ethanol extract *D. caulis* (100, 300, and 500 mg/kg) was orally administered to mice at different doses to confirm its antinociceptive effects on PINP. Behavioral changes were recorded 1 h after *D. caulis* administration. Cold and mechanical allodynia induced by paclitaxel were significantly alleviated in the *D. caulis* 300 and 500 mg/kg group (Figure 3A,B). When the results obtained one hour after the injection of PBS or 100 mg/kg, 300 mg/kg, or 500 mg/kg of *D. caulis* were analyzed by using one-way ANOVA, the F and *p* values in ANOVA interaction were F = 44.81, *p* < 0.0001 and F = 56.92, *p* < 0.0001 for cold and mechanical allodynia, respectively.

### 3.3. Gene Expression of TRPV1 Channel Using qRT-PCR

**Figure 4:**
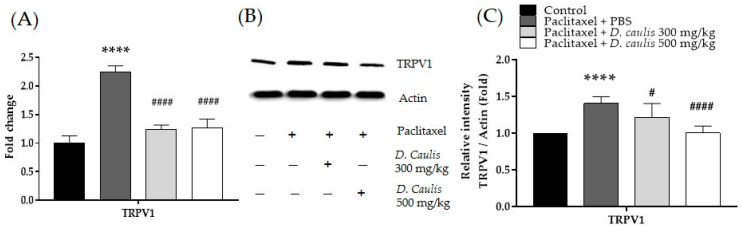
Effect of 300 and 500 mg/kg of *D. caulis* on TRPV1 expression of paclitaxel-induced neuropathic pain in the spinal cord. TRPV1 levels of gene expression (**A**), a representative protein analysis image (**B**), and analyzed relative intensity of TRPV1 protein (**C**). N = 6 per group. **** *p* < 0.0001 vs. control, and ^#^ *p* < 0.05 and ^####^ *p* < 0.0001 vs. paclitaxel + PBS with one-way ANOVA followed by Tukey’s multiple comparison test.

TRPV1 gene expression was confirmed in spinal cord lumbar sections 4–5 from paclitaxel-treated mice. In the paclitaxel-administered group, the expression of TRPV1 increased by approximately 123.73% compared with that in the control group. In contrast, the TRPV1 expression which increased with paclitaxel was significantly decreased by 44.68% and 43.77% in the *D. caulis* 300 and 500 mg/kg groups, respectively (Figure 4A).

Western blotting was performed to elucidate the effect of *D. caulis* on TRPV1 protein expression in paclitaxel-induced pain, and the results were consistent with those of the PCR. Similar to the PCR results, TRPV1 protein expression increased by 40.6% after paclitaxel administration and significantly decreased by 13.4% and 28.6% at 300 and 500 mg/kg of *D. caulis*, respectively (Figure 4B,C).

### 3.4. Identification and Quantification of Rutin and Vicenin-2 in D. caulis

**Figure 5:**
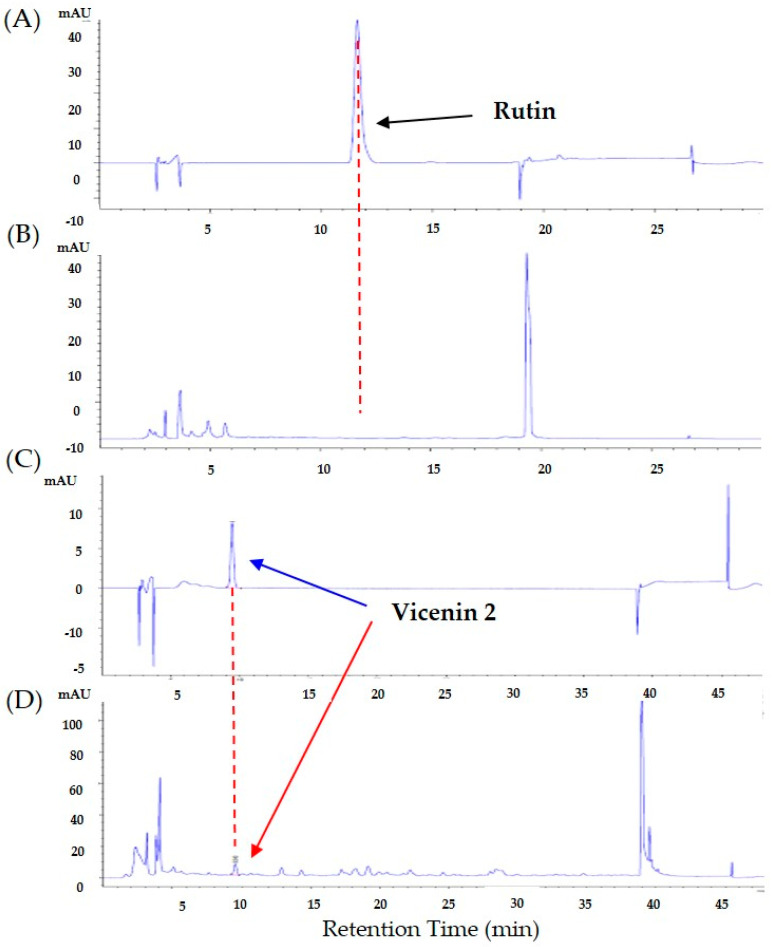
Quantification and identification of vicenin-2 in *D. caulis* by high-performance liquid chromatography (HPLC). HPLC chromatograms of rutin standard (**A**) and *D. caulis* extract for rutin analysis (**B**), and vicenin-2 standard (**C**) and *D. caulis* extract for vicenin-2 analysis (**D**). Black, blue, and red arrows on peaks indicate representative rutin standard, vicenin-2 standard, and vicenin-2 in *D. caulis*, respectively. Retention time and absorbance unit are shown on the *X*-axis and *Y*-axis, respectively.

HPLC analysis was conducted to identify rutin and vicenin-2 as the major components of *D. caulis*. The retention times of rutin and vicenin-2 were approximately 12 and 10 min, respectively (Figure 5A–D). Rutin was not detected, and the UV spectrum and retention of vicenin-2 and vicenin-2 standard solution were consistent. The calibration curve of rutin and vicenin-2 showed linearity in the detector over a range of six concentrations (6.25, 12.5, 25, 50, and 100 μg/mL). The rutin regression equation was y = 17.07092x + 14.48396 and RSQ = 0.99989. The vicenin-2 regression equation was y = 9.23568x + 5.89275 and RSQ = 0.99994. The content of vicenin-2 in *D. caulis* was approximately 0.245%.

### 3.5. Administration of Vicenin-2 Alleviates Paclitaxel-Induced Pain

**Figure 6:**
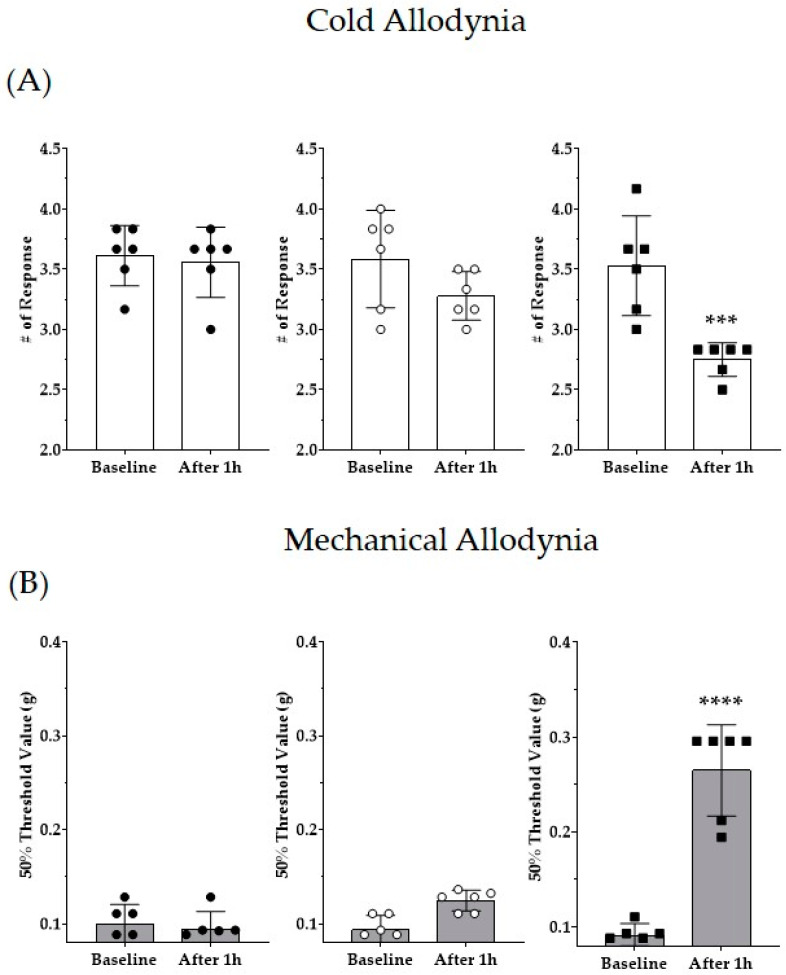
Effects of vicenin-2 on paclitaxel-induced neuropathic pain in mice (**A**,**B**). All groups received multiple injections of paclitaxel (total 8 mg/kg). Doses of 1 and 10 mg/kg of vicenin-2 were injected intraperitoneally in mice. PBS was used as a vehicle for vicenin-2. The black circle, white circle, and black square represent PBS control group, Vicenin-2 1 mg/kg group, and 10 mg/kg group, respectively. N = 6 for each group; *** *p* < 0.001 and **** *p* < 0.0001 vs. control group with paired *t*-test.

Vicenin-2, a major component of *D. caulis*, has been confirmed to exert an antinociceptive effect on PINP. To evaluate the dose-dependent antinociceptive effect of vicenin-2 on PINP, vicenin-2 was intraperitoneally administered to mice at two concentrations (1 and 10 mg/kg). Behavioral responses were recorded before and 1 h after administration of vicenin-2 in the pain-induced mice. The behavioral assessment showed that cold and mechanical allodynia were greatly alleviated in the 10 mg/kg vicenin-2 administration group (Figure 6). When the results obtained one hour after the injection of PBS or 1 mg/kg or 10 mg/kg of vicenin-2 were analyzed by using one-way ANOVA, the F and *p* values in ANOVA interaction were F = 20.76 and *p* < 0.0001 and F = 53.50 and *p* < 0.0001 for cold and mechanical allodynia, respectively.

### 3.6. D. caulis Mimics the Role of TRPV1 Antagonist

**Figure 7:**
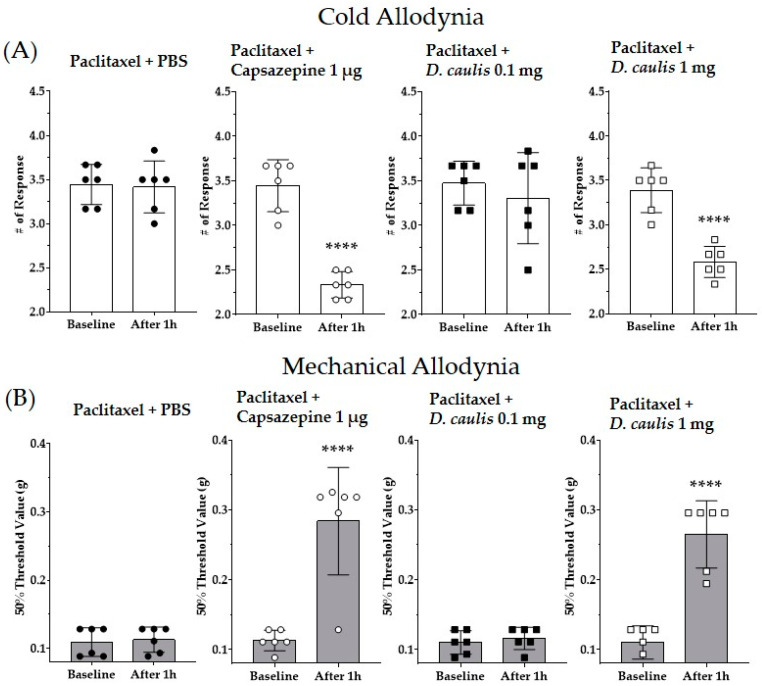
Effects of intrathecal injection of capsazepine and *D. caulis* on paclitaxel-induced neuropathic pain in mice (**A**,**B**). All groups received multiple injections of paclitaxel (total 8 mg/kg). Doses of 1 μg of capsazepine and 0.1 and 1 mg of *D. caulis* were injected intrathecally in mice. PBS was used as a vehicle for *D. caulis*. The black circle, white circle, black square, and white square represent PBS control group, Capsazepine 1 μg, *D. caulis* 0.1 mg, and 1 mg group, respectively. N = 6 for each group; **** *p* < 0.0001 vs. control group with paired *t*-test.

The TRPV1 antagonist capsazepine and *D. caulis* were administered intrathecally, and their antinociceptive effects were compared. In the groups administered with paclitaxel and PBS, there was no change in the pain response even after 1 h. When 1 μg/mouse of capsazepine, a TRPV1 receptor antagonist, was injected intrathecally, cold and mechanical allodynia were significantly alleviated (Figure 7). In addition, *D. caulis* showed excellent antinociceptive effects when administered intrathecally at 1 mg/kg/mouse, with no significant difference from the antinociceptive effect of capsazepine (Figure 7). When the results obtained one hour after the injection of PBS, 1 μg of capsazepine, or 0.1 mg or 1 mg of *D. caulis* were analyzed by using one-way ANOVA, the F and *p* values in ANOVA interaction were F = 17.09 and *p* < 0.0001 and F = 26.50 and *p* < 0.0001 for cold and mechanical allodynia, respectively.

### 3.7. Evaluation of Cell Viability of Vicenin-2 in Caco-2 and RAW 264.7 Cells

**Figure 8:**
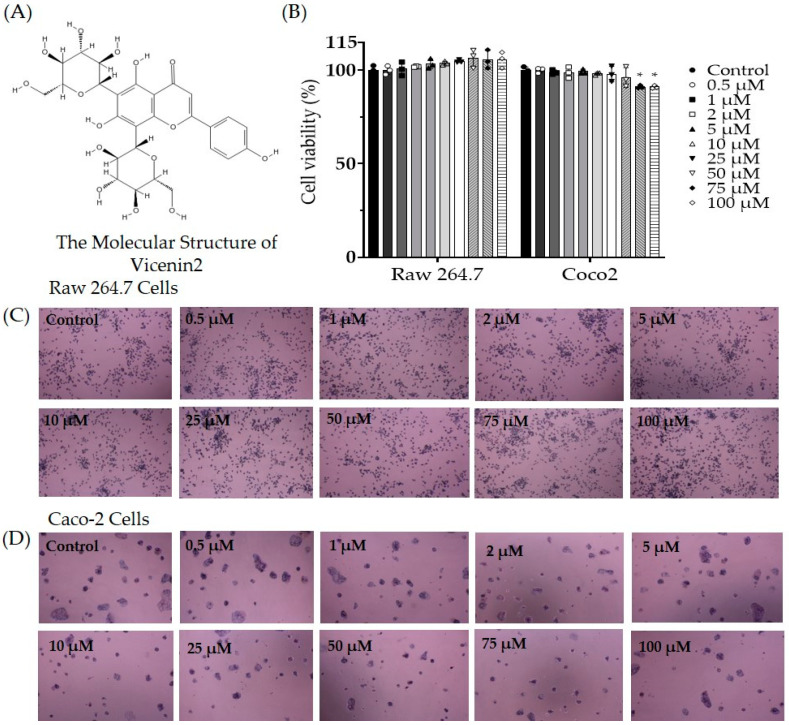
Evaluation of the cytotoxic effect of vicenin-2. The chemical structure of vicenin-2 (**A**). The effect of 10 different doses of vicenin-2 on the viability of RAW 264.7 and Caco-2 cells (**B**) and their morphologies (**C**,**D**). Data were expressed as the mean value ± SD of three independent experiments. N = 3 for each group; * *p* < 0.05 vs. control group with one-way ANOVA followed by Tukey’s multiple comparison test.

To evaluate the cytotoxic effect of vicenin-2, in vitro studies were conducted using human colonic carcinoma Caco-2 cells and non-cancerous RAW 264.7 cells. The cytotoxic effects of vicenin-2 on the viability of the two cell types are presented as the percentage viabilities of three dependent experiments (Figure 8). Vicenin-2 showed a significant cytotoxic effect on Caco-2 cells in concentrations of 75 μM and 100 μM (decreased to 91.10% and 91.05%, respectively). However, vicenin-2 was not cytotoxic in non-cancerous RAW 264.7 cells up to a concentration of 100 μM.

## 4. Discussion

Although paclitaxel has excellent efficacy, only a few injections can cause serious neuropathy, which reduces the patient’s quality of life and may lead to treatment discontinuation. Several analgesic agents are used, but they also induce side effects which limit their wide use [59,60,61]. In this study, 100, 300, and 500 mg/kg *D. caulis* was orally administered to paclitaxel-treated mice. In mice treated with paclitaxel, the pain levels of cold and mechanical allodynia increased significantly and decreased in the *D. caulis* 300 and 500 mg/kg groups. Vicenin-2, the active ingredient of *D. caulis*, was subjected to qualitative and quantitative analyses using HPLC and showed a significant antinociceptive effect at 10 mg/kg. Vicenin-2 did not cause significant cell death in non-cancerous Caco-2 cells up to 50 μM. To determine whether *D. caulis* exhibits an antagonistic effect on TRPV1, capsazepine and *D. caulis* were injected intrathecally, and the same antinociceptive effect was confirmed. In this study, all experiments were conducted in male mice only as some papers have reported that paclitaxel-induced neuropathic pain is not sex specific [57,62]. In addition, no significant difference in body weight change was observed between the sexes after paclitaxel injection [63].

Increased TRPV1 expression in the dorsal horn of the spinal cord has been studied in various types of pain, such as paclitaxel-induced pain (4 mg/kg), sciatic nerve injury, and diabetes-induced neuropathy [64,65,66]. In this study, TRPV1 expression increased in lumbar 4–5 of the spinal cord after paclitaxel administration (total 8 mg/kg), and this upregulation was reduced by *D. caulis*. Furthermore, intrathecal injections of the TRPV1 antagonists capsazepine showed similar analgesic effects to *D. caulis*. Although in this study, the direct effect of *D. caulis* or vicenin-2 on TRPV1 have not been assessed, Kang et al. [67] have reported that 1 h after the intrathecal administration of capsazepine, a significant decrease in TRPV1 protein expression was shown in the spinal cord, suggesting that *D. caulis* may have exerted TRPV1 antagonist-like effects.

Furthermore, early clinical studies have reported that TRPV1 antagonists (AMG-517, 3 mg/kg) increase core body temperature and reduce heat pain recognition in healthy subjects [68]. Preclinical studies have shown that TRPV1 antagonists (XEN-D0501, 5 mg/kg and ABT-102, 4 mg/kg) can block heat hypersensitivity to many proinflammatory agents (e.g., complete Freund’s adjuvant and carrageenan) [69,70]. Preclinical studies using surgical nerve injury models have also reported that TRPV1 expression is altered under chronic pain conditions.

In rodent and human studies, the presence of the TRPV1 protein and mRNA has been confirmed in the spinal cord, hippocampus, hypothalamus, locus coeruleus, striatum, and cerebellum [29]. Focusing on the spinal cord, TRPV1 channels were positive in the presynaptic (from the central terminal of sensory neurons) and postsynaptic regions (from dendrites of spinal cord dorsal horn neurons) and were particularly prominent in areas I and II of the superficial laminae, the first relay regions of the pain sensory pathway [71]. In a study on rhizotomy, a spinal nerve root cutting surgery, histology showed that the postsynaptic TRPV1 expression level was highly dependent on peripheral input, indicating that spinal TRPV1 expression and function can be dynamically regulated in a sensory state. In addition, TRPV1 expression was confirmed in the spinal cord and central nervous system even after rhizotomy, suggesting that TRPV1 receptor proteins show a broad and individual distribution pattern in the central nervous system and that TRPV1 receptors exist in brain regions [72,73]. The antinociceptive effects of TRPV1 antagonists have been studied in several pain models. As reported in these studies, capsazepine, SD-705498, and JNJ-17203212 modestly alleviated osteoarthritic and bone cancer pain related to neuropathic pain [74,75,76]. These results suggest that TRPV1 plays a central role in pain transmission in the spinal cord.

The composition of *D. caulis* was studied using HPLC with electrospray ionization multistage mass spectrometry (HPLC-ESI-MS). The structures of 1 lignan, 6 phenolic acids, and 12 flavonoids have been identified in *D. caulis* [19]. Rutin and vicenin-2 are the major components of *D. caulis*. Rutin has attracted attention as a new antioxidant [77], and vicenin-2 has exhibited anticancer activity by inhibiting the growth of prostate cancer (DU-145, PC-3, and LNCaP cells) cells in vivo and in vitro [78,79] and by inducing apoptosis in HT-29 human colon cancer cells [80]. In addition, vicenin-2 has shown an anti-inflammatory effect by suppressing the protein signaling pathway triggered by TGFβ and inducing H23 cell death through PI3K/Akt/mTOR signaling [81,82]. Vicenin-2 is a major flavonoid component of *D. caulis* [78], and academic consensus suggests that flavonoids can cross the blood–brain barrier (BBB) and reach the central nervous system [83]. Therefore, it has been suggested that vicenin-2 may have a direct effect on the brain and may be used preventively or therapeutically for diseases such as neuropathy.

However, no previous studies have investigated the antinociceptive effects on neuropathic pain induced by anticancer drugs. In this study, rutin was not detected, and vicenin-2 was detected at 0.245%. It has been reported that *D. caulis* varies greatly depending on the harvesting region [84]. Although *D. caulis* and vicenin-2 showed analgesic effects in this study, it cannot be certain that the analgesic effect is entirely dependent on vicenin-2. This is because natural extracts contain many phytochemicals. To confirm the independent effect of vicenin-2, studies should be performed using the Ca^2+^ imaging technique in the TRPV1 overexpression system (e.g., transfected HEK 293 cells) or TRPV1 knock-out mouse. By carefully reviewing the literature relevant to our study, we have found that downregulation of TRPV1 also affects the Ca^2+^ current [85]. This suggests that the gene and protein expression changes demonstrated in our study may also be related to the Ca^2+^ current evoked by spinal TRPV1.

In evaluating the cytotoxicity of vicenin-2 in this study, no cytotoxicity was observed up to 100 μM in non-cancerous cell lines, and toxicity was observed at 75–100 μM in cancer cell lines. In addition, it was reported that vicenin-2 has no cytotoxicity in human dermal fibroblast cells and human umbilical vein endothelial cells up to 400 μM and 100 μM, respectively [80,86]. On the other hand, in human colorectal adenocarcinoma cells (HT-29), which are cancer cells, significant cytotoxicity was observed starting at 25 μM [80]. To date, no adverse effects have been reported for *D. caulis* or vicenin-2. A limitation of this study was the lack of safety data regarding the oral use of *D. caulis*. There is still a lack of safety data owing to limited research, and additional in vivo and cellular studies are needed to confirm its safety. In addition, calcium imaging and electrophysiological studies should be conducted to better understand the correlation between *D. caulis* and TRPV1 channels.

## 5. Conclusions

*D. caulis*, the stem of *D. officinale*, is an important herbal medicine and a natural health food. Collectively, these results suggest that *D. caulis* exerts its antinociceptive effect on PINP by modulating the activity of TRPV1, and the effective substance is vicenin-2. This study provided new insights into the mode of action based on the pharmacological effects of *D. caulis*. Although several biological effects of *D. caulis* have been studied, to the best of our knowledge, this is the first study to suggest an antinociceptive effect of *D. caulis* on anticancer drug-induced neuropathic pain. This suggests that it may be a potentially beneficial ingredient in medicines and food.

## 6. Patent

The content of this article is related to a patent application in Korea (10-2023-0146282).

## Figures and Tables

**Table 1:** Analytical conditions of HPLC for the rutin and vicenin-2 analysis.

Conditions						
Treatment			Rutin			Vicenin-2
Column			Ymc-Triart C18			Ymc-Triart C18
Flow rate			1.0 mL/mL			1.0 mL/mL
Injection volume			10 μL			10 μL
UV detection			275 nm			335 nm
Run time			30 min			48 min
Rutin			Vicenin-2			Flow
Time (min)	Aceto -nitrile	0.1% Phosphoric acid	Time (min)	Aceto -nitrile	0.1% Phosphoric acid	mL/min
0	20	80	0	15	85	1.0
15	20	80	5	15	85	1.0
18	100	0	35	25	75	1.0
23	100	0	37	10	90	1.0
25	20	80	42	10	90	1.0
30	20	80	48	15	85	1.0

## Data Availability

The data presented in this study are available on request from the corresponding author.
